# Spectroscopic imaging of biomaterials and biological systems with FTIR microscopy or with quantum cascade lasers

**DOI:** 10.1007/s00216-017-0574-5

**Published:** 2017-08-29

**Authors:** James A. Kimber, Sergei G. Kazarian

**Affiliations:** Department of Chemical Engineering, Imperial College London, Exhibition Road, London, SW7 2AZ UK

**Keywords:** Fourier transform infrared imaging, Quantum cascade laser, Diagnostics, Scattering, Spatial resolution, Cancer, ATR

## Abstract

Spectroscopic imaging of biomaterials and biological systems has received increased interest within the last decade because of its potential to aid in the detection of disease using biomaterials/biopsy samples and to probe the states of live cells in a label-free manner. The factors behind this increased attention include the availability of improved infrared microscopes and systems that do not require the use of a synchrotron as a light source, as well as the decreasing costs of these systems. This article highlights the current technical challenges and future directions of mid-infrared spectroscopic imaging within this field. Specifically, these are improvements in spatial resolution and spectral quality through the use of novel added lenses and computational algorithms, as well as quantum cascade laser imaging systems, which offer advantages over traditional Fourier transform infrared systems with respect to the speed of acquisition and field of view. Overcoming these challenges will push forward spectroscopic imaging as a viable tool for disease diagnostics and medical research.

**Graphical abstract:**
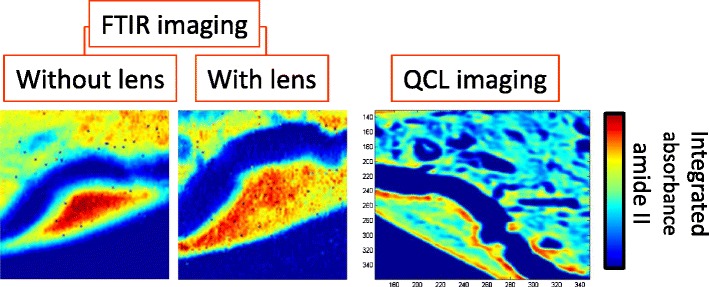
Absorbance images of a biopsy obtained using an FTIR imaging microscope with and without an added lens, and also using a QCL microscope with high-NA objective.

Absorbance images of a biopsy obtained using an FTIR imaging microscope with and without an added lens, and also using a QCL microscope with high-NA objective.

## Introduction

As the understanding of disease progression improves, there is a need to put this knowledge to use in diagnostics, yet acquiring the necessary information may be beyond the scope of routine analytical approaches. The need for rapid and accurate disease diagnostics from biopsy samples, and the effect of newly developed formulations, such as drugs, on cells are of particular recent interest. Current approaches for biopsy sample analysis often rely on the use of staining, which adds a degree of subjectivity from histopathologists, and for studies of cells, molecular labelling can cause misleading results because of changes in the structure or affinity of the target molecule [1, 2]. Vibrational spectroscopic approaches such as Fourier transform infrared (FTIR) spectroscopy are label-free as their chemical specificity allows the differentiation of substances on the basis of chemical content and therefore allows the study of biomaterials and cells without interference from added chemical labelling species [3]. As such, vibrational spectroscopy has attracted much interest for its applicability to study biomaterials and cells, where spectroscopic imaging can offer the possibility of more accurate and faster disease diagnostics, particularly for cancer detection [4–7]. In addition, FTIR spectroscopy can provide new insight into the differentiation of stages of disease, determine tumour margins and enhance understanding of chemical changes at an intracellular level, as well as provide information about the effects of drugs on cells. The wealth of information contained within spectra can also aid in machine learning and classification, leading to statistically rigorous, objective diagnostics [8–10]. This article reports the current state of the art and the challenges within the field of infrared spectral histopathology [7], focusing on specific trends and issues associated with a push for a higher spatial resolution and improving quality of spectral data for analysis of relevant samples.

## FTIR spectroscopic imaging

Since the availability of focal plane array (FPA) detectors in the last two decades, FTIR spectroscopic imaging of biological tissues and cells has become feasible [11, 12]. Imaging has distinct advantages over point mapping, as acquiring an array of spectra over an area takes orders of magnitude less time, and thus also allows the study of dynamic processes. This is particularly advantageous for studies of live cell cultures as there are changes in the morphology and position of cells over time, and it also allows the mapping of large areas (i.e. several square millimetre) of biopsy samples at high resolution (approximately 10 μm) within reasonable timeframes [13, 14]. Infrared microscopes are needed to obtain high-resolution spectroscopic images, and biological samples and systems are typically measured either in transmission mode [15] or in attenuated total reflection (ATR) mode, both of which have distinct advantages and limitations [16]. The quality of the data obtained can be assessed with three metrics: spatial resolution, signal-to-noise ratio (SNR) and presence of spectral artefacts (defined here as changes in absorbance or position of spectral bands due to non-chemical effects). The latter two significantly influence the limit of detection of particular chemical signatures, but the spatial resolution also plays a role as the influence of small regions of interest in the spectra obtained may be averaged out if insufficient spatial resolution is used. Furthermore, in the context of this article, SNR refers to the absorbance noise rather than that of transmittance, as it is the absorption spectrum which contains the spectral information of interest. As such, the absorbance SNR depends on the difference between transmittance spectra, which is in part controlled by sample thickness and not just detector sensitivity or source intensity [17]. Therefore, we discuss here recent trends in achieving high spatial resolution in spectroscopic imaging as well as recent advances for improvement of quality of measured spectra.

FTIR imaging at high spatial resolution using infrared microscopes has been used to study cryosectioned brain tissues for research into Alzheimer’s disease [18], intracellular imaging [19] and discrimination between malaria-infected single cells [20]. The push for high spatial resolutions can come at the expense of the SNR, and for statistical classification, high resolution may not always be necessary. As such, spatial binning can be used to downsample the images so as to find the optimal resolution for computational classification (e.g. studying bronchial biopsy samples for lung cancer diagnostics [8]). When describing spectroscopic imaging at high spatial resolutions, we have to distinguish between optical resolution (i.e. resolving features with sufficient contrast) and spectroscopic spatial resolution (i.e. resolving spectroscopic information from adjacent domains). Recent publications have often reported use of spatial resolution as defined by optical contrast when reporting the performance of infrared microscopes [21]. Briefly, the spatial resolution of a system is determined by the numerical aperture (NA): NA = *n*×sin *θ*, where *n* is the refractive index of the medium between the sample and the objective (e.g. *n*_air_ ≈ 1) and *θ* is the half-angle of the cone of light collected by the objective. This is related to spatial resolution by the Rayleigh/Abbe criterion, where the separation between two resolved points *r* equals 0.61*λ/*NA (0.5λ/NA for the Abbe criterion)*,* where *λ* is the wavelength of light. As an example, the Agilent Cary 620 microscope with a ×15 0.62-NA objective has a maximum theoretical spatial resolution of 2.46 μm at 4000 cm^-1^ and 10.9 μm at 900 cm^-1^. This microscope has high-magnification optics, producing a 2.4 times magnification increase on top of the ×15 objective, giving ×36 magnification, and has a projected pixel size of 1.1 μm when used with a mercury cadmium telluride FPA with 40-μm pixels [18]. However, the theoretically achievable spatial resolution is fundamentally limited by the NA of the optics rather than the projected pixel size. For a discrete detector array, the sampling frequency should also be at least double the desired resolution, so the use of ‘high-magnification’ mode is still beneficial when imaging is performed at the diffraction limit of infrared light [18, 20]. A germanium ATR (Ge-ATR) crystal combined with high-magnification mode allows higher spatial resolutions to be achieved because *n*_Ge_ = 4, producing a measured resolution for optical contrast of 780 nm with a chrome/glass 1951 USAF target [18]. However, the use of high-magnification mode reduces the SNR, and the combination of high magnification with a Ge-ATR crystal has not yet been used for the study of biological samples.

**Fig. 1:**
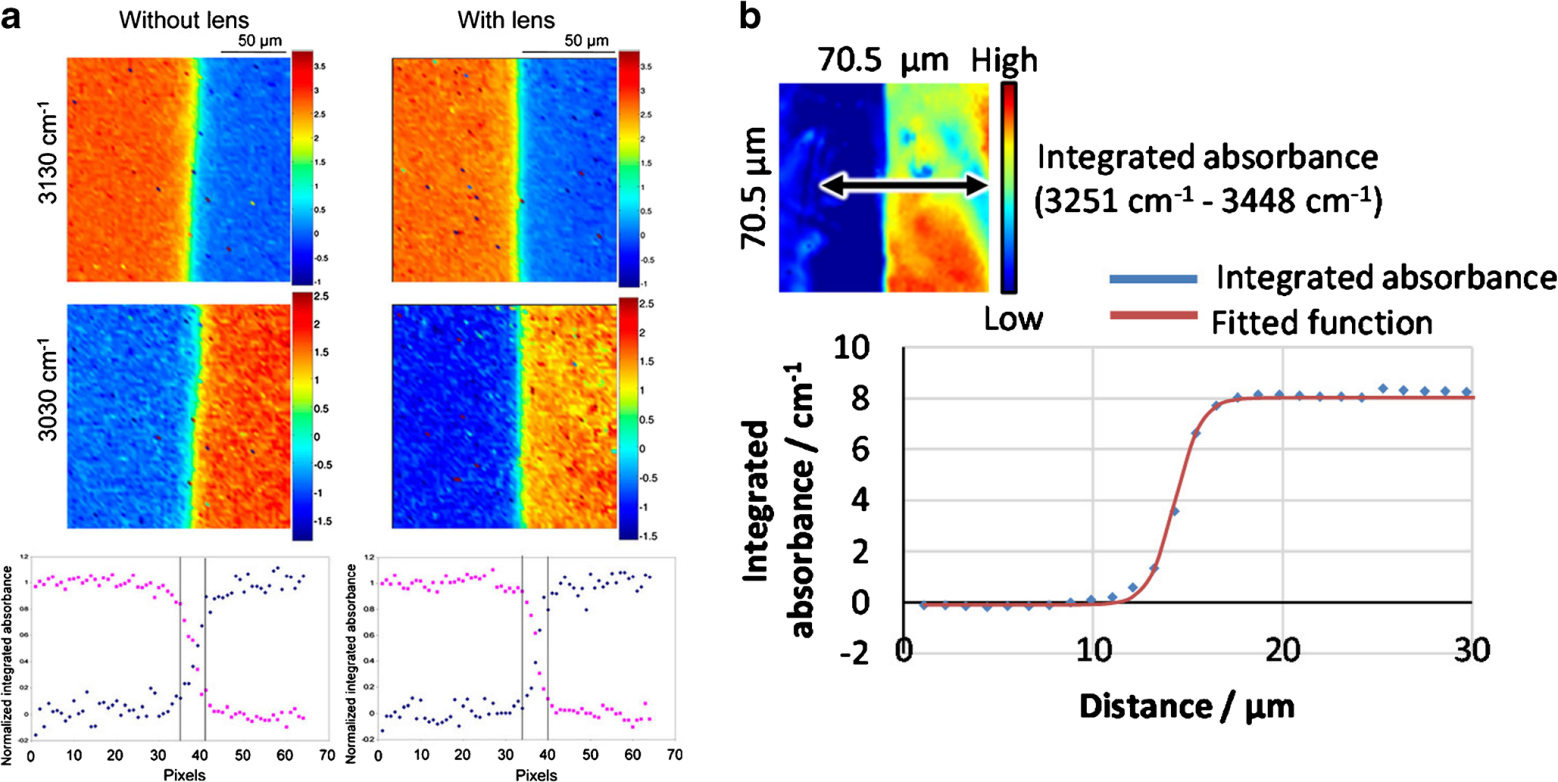
Spatial resolution measured with a polymer interface sample **a** without and with an added lens in transmission mode [24] and **b** with a germanium attenuated total reflection crystal [22]. The measured spatial resolutions were **a** 16 μm and 11 μm respectively and **b** 3 μm across the interface. (**a** Reprinted with permission from [24] copyright 2013 American Chemical Society. **b** Reproduced from [22] with permission from the Royal Society of Chemistry)

The quoted value for the spatial resolution obtained is usually based on the projected pixel size or the Rayleigh/Abbe criterion rather than assessment of actual chemical differences. Furthermore, there is a trend of determining the spatial resolution with optical resolution targets rather than real samples, which especially in the domain of spectroscopic imaging is misleading. In spectroscopic terms, the spatial resolution actually achieved can be defined as the extent of the spectral difference between two chemically distinct regions separated by a sharp interface, as resolving the chemical differences between spatial regions is the objective of spectroscopic imaging rather than artificial resolution targets [22]. Kazarian and Chan [23] proposed the assessment of spatial resolution in spectroscopic imaging systems on the basis of the distance required for a step function across a sharp interface, which in their study was created with two immiscible polymers, to change from approximately 95% to approximately 5% of its maximum value at one of these polymer domains. They showed that the achievable spatial resolution in the spectroscopic images obtained (i.e. the distance for the change of absorbance of a spectral band across an interface) is three times lower than the resolution obtained with a USAF target [24]. We believe that assessment of the spatial resolution in this way is of particular importance for biological samples as their chemically distinct regions will not have such sharp refractive index changes like a USAF target. This knowledge would eliminate the possibility of spectral ‘contamination’, for example between adjacent cells, by ensuring they are located further apart than the determined spectroscopic imaging resolution. Therefore, a simple, practical way to assess the achieved spatial resolution for spectroscopic imaging would be to use a polymer interface as recently demonstrated [22–24] and shown in Fig. 1.

**Fig. 2:**
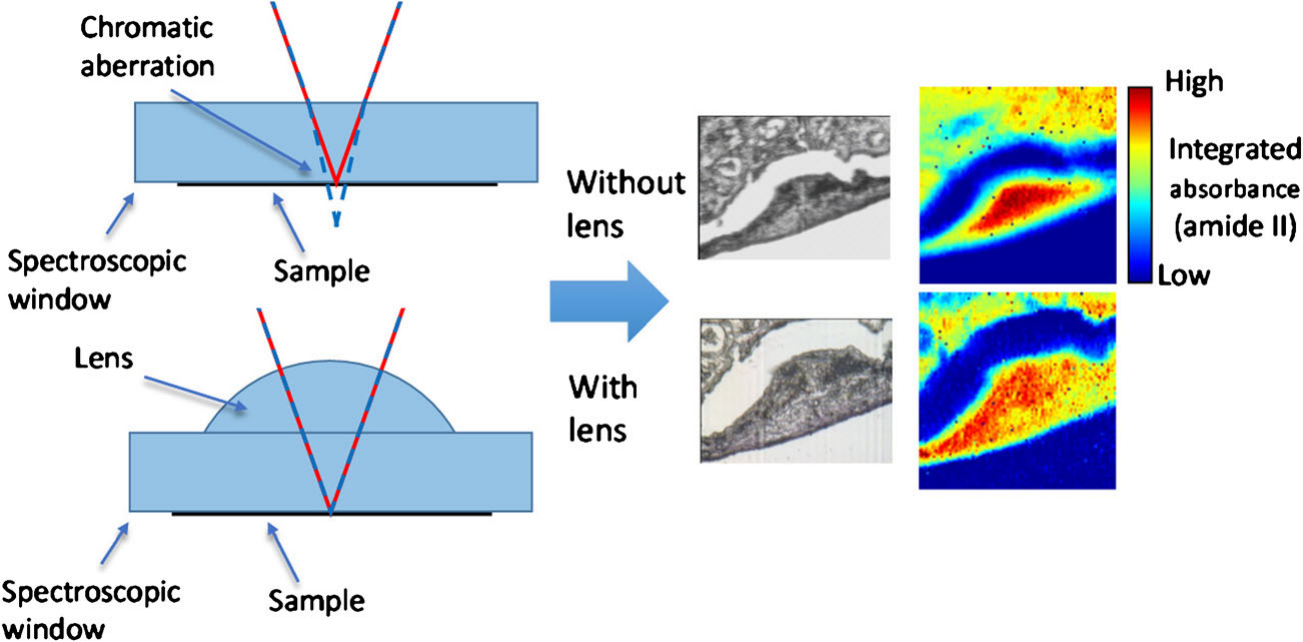
The added lens approach for studying oesophagus biopsy samples. The lens and window form a pseudo hemisphere which removes chromatic aberration, reduces scattering and provides additional ×1.4 magnification

Another method to increase the spatial resolution is to use the substrate on which the biopsy samples or cells are situated as part of an immersion objective. Chan and Kazarian [24] combined a transmission cell with 4-mm-thick CaF_2_ windows with added lenses to form a ‘pseudo hemisphere’. The objective of their work was to correct for chromatic aberration as the lenses would ensure all wavelengths of infrared light were in focus at the sample when it was sandwiched between two windows. Without the added lenses, infrared light is refracted at the planar windows as a function of wavelength, requiring the selection of a particular wavelength to be in focus, with the others defocused to some degree [24]. The added lenses also enhanced the spatial resolution by 1.4 times because of the refractive index of CaF_2_, which changes the NA, and is thus a real magnification (Fig. 1a). They are especially useful for studying live cells in transmission mode which must be enclosed in a microfluidic device between infrared-transparent windows, and therefore without the lenses, refraction is unavoidable [25]. For measurement of biopsy samples, the microscope objective is usually above the tissue, supported by the window on its underside, and thus only the illuminating infrared light passes through the window rather than the light collected by the objective. However, deliberate inversion of the window and use of the added lens provides the additional ×1.4 magnification, and combined with a suitable mechanism allows the mapping of large areas as the lens can be fixed within the optical axis and the sample and window can be slid with use of a microscope stage [22]. This added lens approach thus allows ×1.4 magnification to be obtained without any modifications needed to the existing optics within the microscope (Fig. 2).

**Fig. 3:**
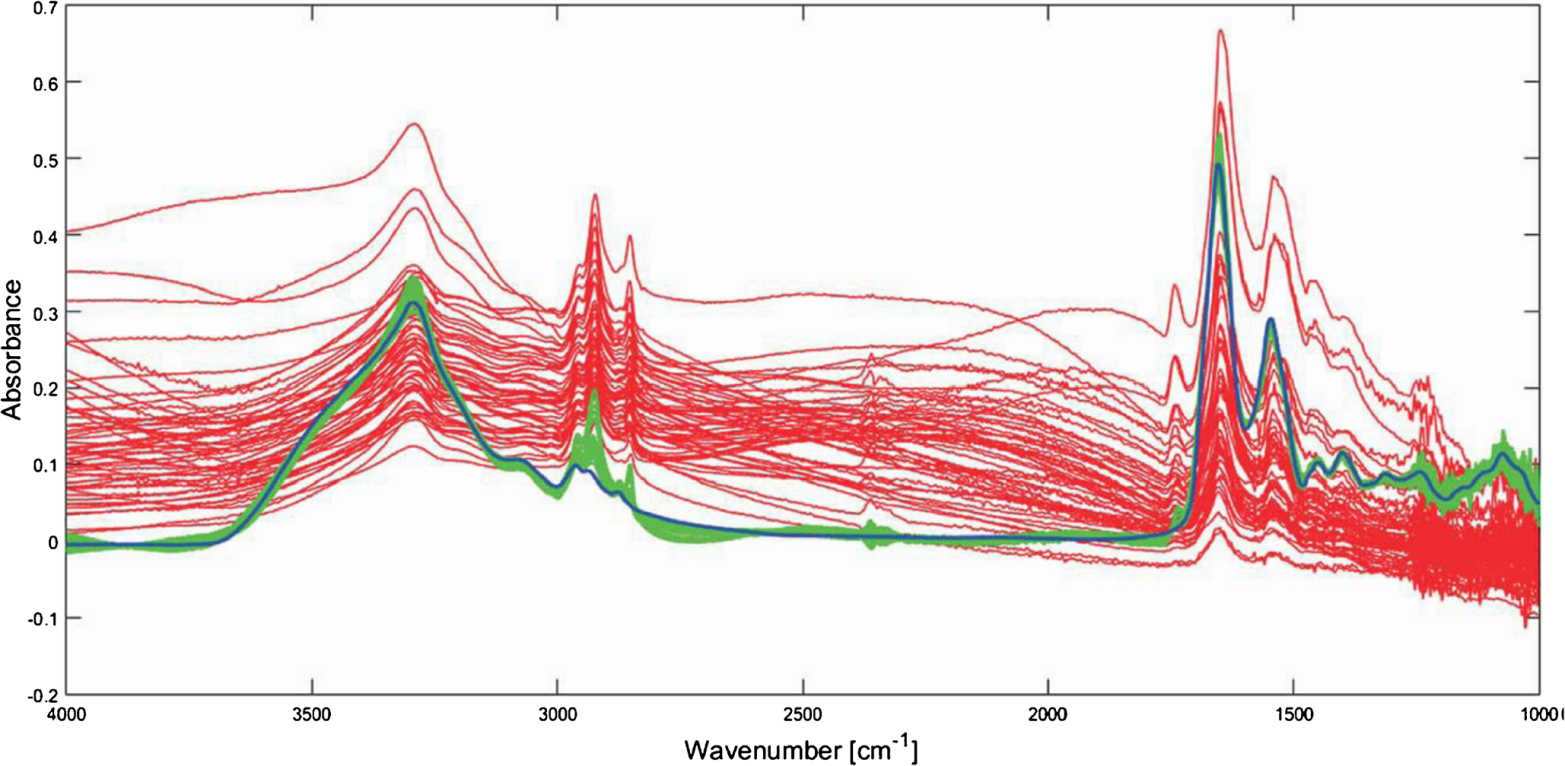
Measured spectra of lung cancer cells (*red*) with Mie scattering are corrected by an improved resonant Mie scattering/extended multiplicative signal correction scattering algorithm to produce the *green spectra* [28]. (Reproduced from [28] with permission from the Royal Society of Chemistry)

A further benefit of the added lens approach is a reduction in scattering, a phenomenon that manifests itself around the amide I band, causing an apparent shift [26]. This resonant Mie scattering results from features of the sample that are similar in size to the wavelength of infrared light. This is problematic for biopsy samples and cells, but the use of a CaF_2_ window and lens results in closer refractive index matching, reducing the intensity of the resonant Mie scattering [22]. Computational algorithms can also be used to remove Mie scattering, and a method developed by Kohler et al. [27] used a metamodel of Mie extinction efficiencies simulated for the scattering and absorption of infrared radiation at spheres with a range of radii and refractive indices. The simulated spectra are summarized in a principal component analysis model and implemented in an extended multiplicative signal correction model for retrieval of pure absorption spectra, removing the scattering. Improvements to this approach by Bassan et al. [28] resulted in a resonant Mie scattering/extended multiplicative signal correction algorithm that can be used to remove this phenomenon [29], and further refinements of the algorithm have resulted in speed and quality improvements (Fig. 3).

Resonant Mie scattering can also be completely removed by use of a Ge-ATR crystal, whilst providing a high spatial resolution (measured across a polymer interface) of 3 μm (Fig. 1b) [22]. ATR mode relies on an evanescent wave at the surface of the crystal which interacts with the sample. The degree to which this wave interacts with the sample, also known as the *depth of penetration* (where the electric field intensity of the wave falls to e^-1^ its strength at the surface) and the *effective path length* (where the same material of a certain thickness measured in transmission mode would produce the same absorbance values), depends on the refractive index of the crystal and sample, the angle of incidence of infrared light entering the crystal and the wavelength of infrared light. For a Ge-ATR crystal in an infrared microscope measuring biological samples, the depth of penetration at 1000 cm^-1^ is approximately 1 μm. This means that the sample must be in intimate contact with the crystal, and for biopsy samples this is not an issue because of their deformability. There is the potential for damage to the sample and cross-contamination when mapping is performed with a Ge-ATR crystal as it is a repeated contact-based measurement as opposed to transmission mode, where the sample is in constant contact with the substrate.

The choice of substrate is also important with respect to cost and also performance, as different materials have different transmission spectra; for example, CaF_2_ attenuates infrared frequencies below 1200 cm^-1^, whereas BaF_2_ substrates do not, yet BaF_2_ is toxic to cells and slightly soluble in water unless its surface is modified [30]. For CaF_2_, bands corresponding to nucleic acids, collagen and glycogen [9] are attenuated or lost, and for disease diagnostics, the spectral band changes in this region are important [8], but the degree of attenuation is a function of substrate thickness, and use of thin windows can alleviate this problem. For biopsy samples, inexpensive transflection slides can also be used, but these have additional constraints on the sample thickness because of the infrared light passing twice through the material, and potentially uneven surfaces giving rise to standing-wave effects, resulting in absorbance values that are not representative of the sample [31]. By contrast, a Ge-ATR crystal is particularly suitable for studying live cells in an aqueous environment because of its non-toxic nature, relatively flat transmittance across the whole mid-infrared range, high refractive index giving rise to high spatial resolution, and ability to remove scattering. Furthermore, the size of the evanescent wave defines the effective path length, whereas in transmission mode, aqueous systems typically require a path length of approximately 10 μm or less to avoid saturation of measured absorbance by water. Consequently, cells must fit within this space, somewhat restricting the mass transfer of materials to the sides of the cell as the top and bottom surfaces may be in contact with the spectroscopic windows, unless care is taken to preseed the cells on one window so this is avoided. These conditions could place cells under stress, affecting their normal reactions to external stimuli, and are completely avoided with the use of a Ge-ATR crystal, as only the bottom part of the cell is in contact with a surface [16].

## Imaging with quantum cascade lasers

Infrared spectroscopy based on quantum cascade lasers (QCLs) as a source of infrared radiation use discrete-frequency tuneable lasers which produce mid-infrared light several orders of magnitude brighter than that from a silicon carbide heating element or synchrotron. QCL spectrometers are also more compact and less sensitive to vibrations than benchtop FTIR spectrometers with moving mirror interferometers. The high spectral power density of QCLs is ideal for use in microscopes, where samples need to be illuminated by a concentrated beam, and allow thermoelectrically cooled bolometer array detectors (480 × 480 pixels with 17.5-μm^2^ pixel size) to be used, compared with liquid-nitrogen-cooled FPA detectors with 128 × 128 pixels and 40-μm^2^ pixel size that are used for FTIR imaging. These factors make QCLs attractive for spectroscopic imaging within clinical environments, and they have proven to be capable of measuring large areas of a single biopsy sample or tissue microarrays within a relatively short time. For example, spectroscopic imaging of a 1mm-diameter tissue sample would take between 5 and 6 h with a comparable FTIR imaging microscope system [1], whereas by use of the discrete nature of a QCL microscope system to measure only a small number of frequencies needed for disease classification (e.g. 31), a similar sample of the same diameter can be measured in less than 6 min. With an FTIR spectrometer, increasing the moving mirror speed, changing the interferogram sampling interval and lowering the spectral resolution are methods to decrease image acquisition time, but this still produces a large amount of data in the form of interferograms that must be recorded and processed. As such, the scope for data reduction without significant loss of classification performance when discrete-frequency QCL systems are used allows much larger spatial areas to be analysed than previously feasible because of fast data acquisition, facilitating larger high-throughput studies of multiple samples.

**Fig. 4:**
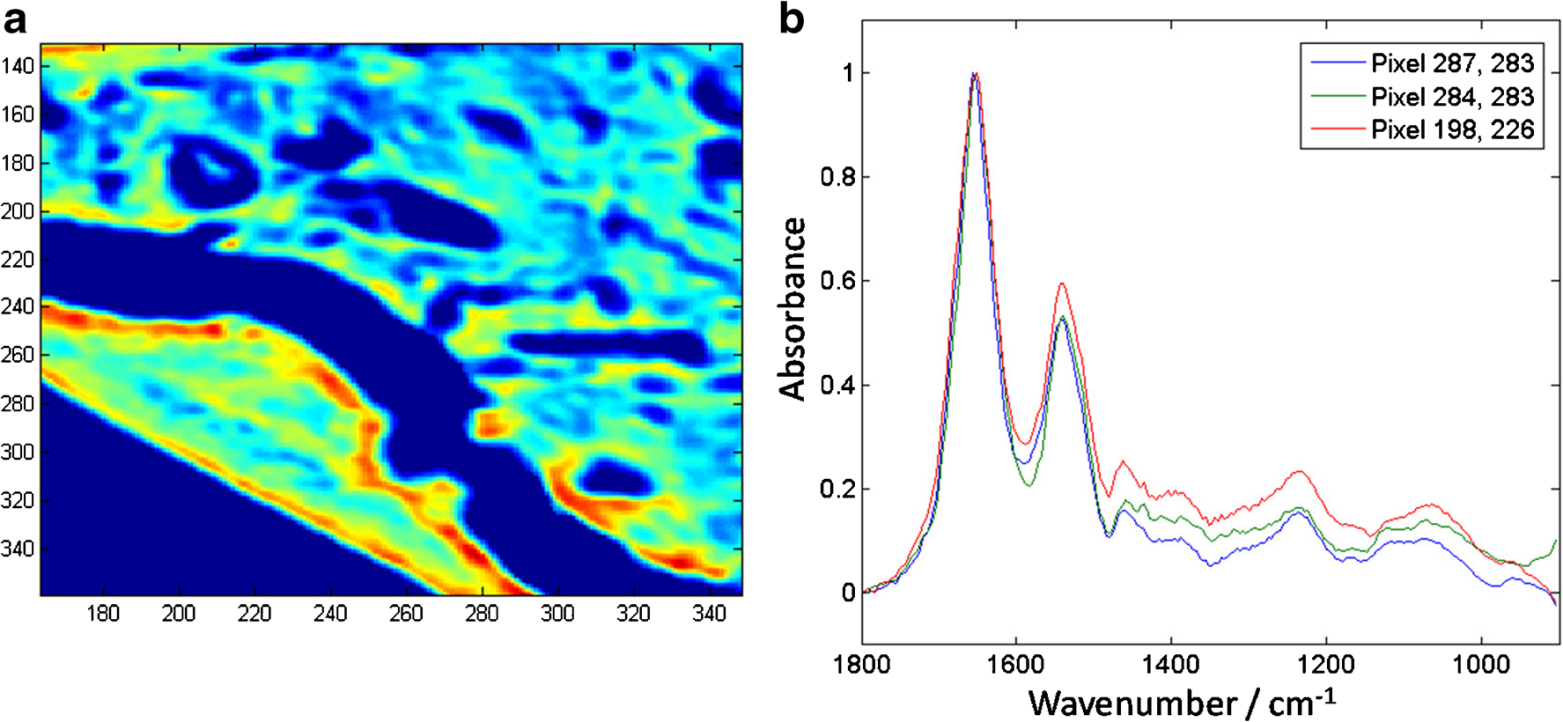
Data obtained from a Daylight Solutions Spero microscope where the same sample as in Fig. 2 was measured, showing **a** the spectroscopic image of peak height at 1540 cm^-1^ and **b** example extracted spectra from different pixels. All spectra were collected with 0.5-cm^-1^ steps before application of a moving average of 8-cm^-1^ width, and downsampling to 4 cm^-1^, where the spectra shown in **b** have been normalized with respect to the amide I band

The theoretical spatial resolution, as determined by the Rayleigh criterion, of QCL microscopes is also comparable with that of FTIR microscopes, where the Daylight Solutions Spero system uses a ×12 0.7-NA objective to produce a projected pixel size of 1.4 μm—similar to that of the Agilent Cary 620 in high-magnification mode. As such, QCL systems are capable of acquiring spectroscopic images at the diffraction limit. An example of the high spatial resolution and spectral quality can be seen in Fig. 4, where the same sample as in Fig. 2 was measured with a Spero system. Recent publications further demonstrate the use of such a system for histopathology (e.g. adenocarcinoma colorectal tissue [32]) and high-speed scanning of an entire liver biopsy (1.8 × 1.2 cm^2^), collecting 12.4 million sparse-frequency spectra (i.e. only those frequencies necessary for classification) within 16.2 min [33]. These interesting biomedical applications focused on demonstrating the advantages of QCLs for biomedical applications, such as fast data acquisition, the flexibility of tuneable laser sources and use of room-temperature detectors, which may be useful for practical implementation of such systems with the aim of their translation to clinics.

However, current QCL systems are able to measure wavelengths only within the range from 1800 to 900 cm^-1^and thus spectra at higher wavenumbers (i.e. up to 4000 cm^-1^) cannot be obtained. Furthermore, a single QCL can be tuned from its centre frequency by only approximately ±110 cm^-1^, so a spectrometer covering the entire fingerprint region would need at least four QCL modules [34]. Because of the coherent source of infrared radiation, spectroscopic images from wide-field QCL systems can also contain artefacts in the form of interference or fringe patterns, although the addition of a diffuser plate in the beam path reduces these artefacts [35]. For QCL scanning systems, the presence of a coherent source is not a problem; hence, a scanning system is another way to overcome this issue [36]. The factors affecting the quality of spectra from a QCL system also differ from those of an FTIR spectrometer, as increasing the number of scans for a QCL system may not increase the SNR. This is due to instability within the QCL itself, and Yeh et al. [37] measured the output of a QCL with a mercury cadmium telluride FPA and noted that despite their being able to acquire a 128 × 128 spectral images at 0.25-cm^-1^ resolution within 3 s, spectral noise significantly increased, resulting from this rapid scanning, and was especially present at the frequency crossover regions of the QCLs, although this can be reduced with postprocessing filters. Power fluctuations and tuning mismatch between the background and sample scans contributed to this, and as such, assuming linearity of a frequency sweep was found to be incorrect, and that greater stability is gained by discrete stepping of the frequencies but with a significant increase in scan time. However, improvements in QCL stability result in the SNR increasing in the same way as for an FTIR spectrometer (SNR ∝ (no. scans)^0.5^) [17]. In spite of these limitations, the advantages of discrete-frequency imaging are clear in the time required to scan large areas, where an acquisition speed more than 1000 times greater than with an FTIR spectrometer can be obtained, and recent improvements in QCL scanning rates and acquisition stability have been noted, where rapid-scan microoptoelectromechanical QCLs can acquire spectra within 20 ms [38].

An exciting application of QCLs for high-throughput analysis of biofluids has recently been introduced [39] which offers new opportunities for medical diagnosis using such discreet-frequency infrared technology. Hughes et al. [39] found that application of discrete-frequency imaging to study the features of the amide I and amide II bands from dried human blood serum can speed up the classification between non-cancerous and cancerous samples significantly [39]. Specifically, they highlight how recent advances have reduced acquisition times of a single serum image spot (approximately 480 × 480 pixels) with 199 frequency data points between 1800 and 1000 cm^-1^ from 11.3 to 4.3 min, and by careful choice of fourteen or even nine discrete frequencies (whilst maintaining good classification performance), an acquisition time of 56 s or 36 s respectively is possible. Other recent developments and applications of this method were described by Wrobel et al. [40]. They note the speed of image acquisition is 100 times higher than that of the fastest FTIR imaging systems, and cite several examples of systems that benefit from this speed (e.g. spectroscopic imaging of living microorganisms [41]).

## Outlook

Advances in spectroscopic imaging of biological systems and samples and the focus on disease diagnostics make compelling cases for its use in spectral histopathology within a clinical environment and the study of drugs within cells. The wealth of information available in spectra and the extraction of key spectroscopic changes from samples in a non-destructive way allows classification between cells and disease states, and has the potential to enhance the sensitivity and specificity above those of currently used methods. The challenges of obtaining quality data from biopsy samples and cells in terms of good SNR, artefact-free spectra and high acquisition speed are being addressed in a variety of ways, from computational algorithms and novel optics to more powerful sources of infrared and the development of compact QCL-based imaging spectrometers. Combining these developments in spectroscopic imaging into the design of new microscopes and instruments, such as the use of a QCL source with a high-magnification microscope equipped with added lenses and/or an ATR crystal, would allow the acquisition of higher-resolution, higher-quality spectroscopic images than the current state of the art. This, along with larger data sets and applications of machine learning, brings closer the prospect of routine ‘digital histopathology’ [36] and spectral cytopathology, and the formation of multitype relational data sets comprising data from a combination of approaches, such as FTIR, Raman and fluorescence imaging, would also significantly advance these areas of research. Nevertheless, spectrometers based on the recent advances discussed here are still some time away from being used in clinics, due in part to the need for validation of the practical approach and data processing algorithms. This is why research in this area going beyond the state of the art is needed, bringing its outcomes for the benefits of improved diagnostics and treatment in health care. In summary, label-free FTIR spectroscopic imaging and QCL-based imaging offer the possibility of addressing clinical challenges by providing enhanced disease diagnostics, particularly for cancer detection and analysis, as spectroscopy has a potential to provide new insight into the differentiation of stages of disease and enhance our understanding of chemical changes at an intracellular level.
